# VEZF1–guanine quadruplex DNA interaction regulates alternative polyadenylation and detyrosinase activity of VASH1

**DOI:** 10.1093/nar/gkaa1092

**Published:** 2020-11-24

**Authors:** Lin Li, Preston Williams, Zi Gao, Yinsheng Wang

**Affiliations:** Department of Chemistry, University of California, Riverside, CA 92521, USA; Department of Chemistry, University of California, Riverside, CA 92521, USA; Department of Chemistry, University of California, Riverside, CA 92521, USA; Department of Chemistry, University of California, Riverside, CA 92521, USA

## Abstract

Vascular endothelial zinc finger 1 (VEZF1) plays important roles in endothelial lineage definition and angiogenesis. Vasohibins 1 and 2 (VASH1 and VASH2) can form heterodimers with small vasohibin-binding protein (SVBP) and were recently shown to regulate angiogenesis by acting as tubulin detyrosinases. Here, we showed that VEZF1 binds directly with DNA guanine quadruplex (G quadruplex, G4) structures *in vitro* and in cells, which modulates the levels of the two isoforms of VASH1 mRNA. Disruption of this interaction, through genetic depletion of VEZF1 or treatment of cells with G4-stabilizing small molecules, led to increased production of the long over short isoform of VASH1 (i.e. VASH1A and VASH1B, respectively) mRNA and elevated tubulin detyrosinase activity in cells. Moreover, disruption of VEZF1-G4 interactions in human umbilical vein endothelial cells resulted in diminished angiogenesis. These results suggest that the interaction between VEZF1 and G4 structures assumes a crucial role in angiogenesis, which occurs through regulating the relative levels of the two isoforms of VASH1 mRNA and the detyrosinase activity of the VASH1-SVBP complex. Together, our work revealed VEZF1 as a G4-binding protein, identified a novel regulatory mechanism for tubulin detyrosinase, and illustrated that the VEZF1- and VASH1-mediated angiogenesis pathways are functionally connected.

## INTRODUCTION

The blood vasculature is essential in embryonic development and in the homeostasis of adult tissues (1), and angiogenesis assumes indispensable functions in wound healing and cancer (2,3). Multiple transcription factors are known to be crucial in angiogenesis, including, among others, vascular endothelial growth factor (VEGF) and vascular endothelial zinc finger 1 (VEZF1) (4). The importance of VEZF1 in angiogenesis is manifested by the fact that *Vezf1*^−/−^ mice exhibit defects in vascular remodeling and loss of vascular integrity (5). In addition, VEZF1 regulates many genes involved in angiogenesis, including endothelin-1, cited2, metallothionein-1 and stathmin/oncoprotein 18 (6–9). A previous chromatin immunoprecipitation followed by sequencing (ChIP-seq) analysis revealed >30 000 VEZF1 binding sites in chromatin (10), where VEZF1 binding sites are strongly associated with sites of RNA polymerase II (RNAP II) pausing (10). Moreover, VEZF1 was found to modulate the splicing of DNMT3B mRNA (11). However, the molecular determinant through which VEZF1 regulates RNAP II pausing and alternative splicing remains undefined.

Aside from VEZF1, vasohibins 1 and 2 (VASH1 and VASH2) also assume important regulatory functions in angiogenesis. These two proteins form heterodimers with SVBP, and they were recently discovered as the long-sought tubulin carboxypeptidases for the removal of the tyrosine residue located on the C-termini of α- and β-tubulins (12,13). Dynamic detyrosination and tyrosination, the latter of which is mediated by tubulin tyrosine lyase (14), of α- and β-tubulins allow for maintaining microtubule homeostasis as well as enabling angiogenesis and neuron differentiation (12,13). *VASH1*, a VEGF target gene in human umbilical vein endothelial cells (HUVECs) (15), is known to undergo alternative splicing (16,17). Nevertheless, it remains unclear how alternative splicing of VASH1 is regulated and how this alternative splicing modulates tubulin carboxypeptidase activity.

Herein, we uncovered VEZF1 as a guanine quadruplex (G4)-binding protein, and revealed its binding to G4 structure at the junction of VASH1A and VASH1B. We also showed that this binding modulates the alternative polyadenylation of VASH1 mRNA and cellular detyrosinase activity, thereby regulating angiogenesis. Hence, our work also unveiled the functional coupling of the two important regulatory pathways of angiogenesis.

## MATERIALS AND METHODS

### Cell lines

HEK293T (293T) cells were purchased from ATCC (Manassas, VA, USA) and maintained in Dulbecco's modified Eagle's medium (DMEM, Life Technologies, Carlsbad, CA) supplemented with 10% fetal bovine serum (FBS, Invitrogen, Carlsbad, CA, USA) and 100 unit/ml penicillin/streptomycin. HUVECs were purchased from Lonza Bioscience (C2519A, Basel, Switzerland). HUVECs were maintained in EBM-2 Basal Medium (CC-3156, Lonza Bioscience) with EGM-2 SingleQuots Supplements (CC-4176, Lonza Bioscience). All cells were cultured at 37°C in a humidified incubator with 5% CO_2_.

### Electrophoretic mobility shift assay (EMSA)

The coding sequence of human VEZF1 was inserted into the pRK7 vector with 3 tandem repeats of Flag epitope tag on the carboxyl terminus. The plasmids were transfected into HEK293T cells using TransIT-2020 (Mirus Bio, Madison, WI) and, at 24-h later, the cells were lysed in CelLytic M Cell Lysis Reagent (Sigma, St. Louis, MO) containing 1× protease inhibitor cocktail (Sigma). The Flag-tagged protein was purified using Anti-Flag M2 Affinity Gel (Sigma) and eluted with 3× Flag peptide (NC0792928, Thermo Fisher Scientific, Waltham, MA). Proteins were quantified using Bradford Protein Assay Kit (Bio-Rad, Hercules, CA), and their purities verified by SDS-PAGE analysis.

For protein-DNA binding, 25 nM 5′-TAMRA-labeled DNA was incubated with different concentrations of VEZF1 protein in a buffer containing 10 mM Tris-HCl (pH 7.5), 100 mM KCl, 10 μM ZnCl_2_, 1 mM DTT and 3% glycerol on ice for 30 min. The samples were then loaded onto a 6% polyacrylamide gel in TBE buffer (40 mM Tris–HCl, pH 8.3, 45 mM boric acid and 1 mM EDTA) at 4°C. The gel was run at 120 V and at 4°C for 30 min, and was subsequently imaged with Odyssey Imaging Systems (LI-COR Biosciences, Lincoln, NE, USA).

### Chromatin fractionation and Western blot

Chromatin fractionation was performed as described (18). Briefly, the chromatin fraction was isolated using a cytoplasmic lysis buffer (10 mM Tris–HCl, pH 8.0, 0.34 M sucrose, 3 mM CaCl_2_, 2 mM MgCl_2_, 0.1 mM EDTA, 1 mM DTT, 0.5% NP-40 and a protease inhibitor cocktail), a nuclear lysis buffer (20 mM HEPES, pH 7.9, 1.5 mM MgCl_2_, 1 mM EDTA, 150 mM KCl, 0.1% NP-40, 1 mM DTT, 10% glycerol and a protease inhibitor cocktail) and a chromatin isolation buffer (20 mM HEPES, pH 7.9, 1.5 mM MgCl_2_, 150 mM KCl, 10% glycerol, a protease inhibitor cocktail and 0.15 unit/μl benzonase). After separation on a 10% SDS-PAGE, the proteins were transferred onto a nitrocellulose membrane (Bio-Rad). After blocking with blotting-grade blocker (Bio-Rad), the membrane was incubated in a solution containing primary antibody and 5% BSA for 2 h, and then incubated in a 5% blotting-grade blocker containing the HRP-conjugated secondary antibody. The Western blot signal was detected using ECL Western blotting detection reagent (Amersham, Little Chalfont, UK).

Primary antibodies used in this study included histone H3 (Cell Signaling Technology, Danvers, MA, 9715S; 1:10 000 dilution), VEZF1 (Santa Cruz Biotechnology, Dallas, TX, SC-365560; 1:2000), VASH1 (Santa Cruz Biotechnology, SC-36554; 1:2000), HA (Santa Cruz Biotechnology, SC-57592; 1:5000), detyrosinated tubulin (Millipore Sigma, Burlington, MA, AB3201; 1:3000) and tubulin (Santa Cruz Biotechnology, SC-32293; 1:5000).

### Chromatin immunoprecipitation-quantitative PCR (ChIP-qPCR)

ChIP experiments were conducted as previously described with a few modifications (18). For ChIP of VEZF1, HEK293T cells were transfected with pRK7-VEZF1–3× Flag plasmid for 12 h, and then treated with or without 20 μM pyridostatin (PDS) and or 5 μM 5,10,15,20-tetra-(*N*-methyl-4-pyridyl)porphyrin (TMPyP4) for another 12 h. Approximately 2 × 10^6^ cells were cross-linked with 1/10 volume of freshly prepared 11% formaldehyde in water at room temperature for 10 min, and quenched with 125 mM glycine for 5 min. After washing with 1× PBS for three times, the cells were resuspended in PBS. Chromatin was sheared using a Covaris S220 sonicator at 4°C for 6 min with a peak incident power of 140, a duty cycle of 5%, and 200 cycles per burst. ChIP was performed using anti-Flag antibody (Cell Signaling Technology, 2368S), and DNA was eluted and purified using QIAquick PCR Purification Kit (Qiagen, Hilden, Germany). Quantitative PCR was conducted using the primers listed in Supplementary Table S1.

For ChIP of BG4, chromatin was immunoprecipitated using BG4 antibody, which was purified following published procedures (19). Anti-RNAP II S2P antibody (Abcam, Ab5095) and Protein A/G Plus-Agarose (Santa Cruz Biotechnology) were employed for the ChIP analysis of RNAPII-S2P. After purification of the antibody-enriched DNA fragments, quantitative PCR was performed using the primers listed in Supplementary Table S1.

For overlapping analysis, each peak in the two BED files were compared, and those peaks overlapped in the two ChIP-seq datasets (i.e. VEZF1 and BG4 ChIP-seq) by at least 1, 8 or 30 base pairs are considered overlapping peaks with custom script (Supplementary Tables S2-S3).

### Real-time quantitative PCR (RT-qPCR)

RT-qPCR was conducted as previously described (18). Total RNA was extracted with Omega Total RNA Kit I (Omega, Norcross, GA, USA) and quantified. Reverse transcription was performed using M-MLV Reverse Transcriptase (Promega, Madison, WI, USA) for cDNA synthesis. RT-qPCR was carried out using iQ SYBR Green Supermix (Bio-rad) on a CFX96 RT-qPCR detection system (Bio-Rad). Primers used for RT-qPCR are listed in Supplementary Table S1.

### *In vitro* transcription assay

The DNA template for the in-vitro transcription assay was prepared by inserting the G4 region derived from the junction of VASH1A and VASH1B, or the corresponding mutated sequence that was not able to fold into G4 structure, into the XhoI and XmaI sites of a pRK7-GFP plasmid, where the insert was placed at 824 bp downstream of the CMV promoter. Another DNA fragment was inserted at the XmaI and EcoRI sites downstream of the G4-containing insert. The plasmid was subsequently linearized with SpeI and KpnI. The transcription experiment was performed in a 25-μl solution containing 10 μl HeLa nuclear extract (Promega, Madison, WI, USA), 1 μl HeLa nuclear extract transcription buffer (Promega, Madison, WI, USA), 3 mM MgCl_2_, 0.4 mM rATP, 0.4 mM rGTP, 0.4 mM rCTP, 16 μM rUTP, 200 ng of the aforementioned linear DNA fragment and 1 μl [α-^32^P]UTP (3000 Ci/mmol, 10 mCi/ml). The mixture was incubated at 30°C for 60 min. The reaction was terminated by adding 175 μl Stop solution (Promega, Madison, WI, USA). The resulting RNA was extracted from the mixture using phenol, precipitated with ethanol, and resolved on a 6% polyacrylamide gel containing 7 M urea.

### Angiogenesis assay

The angiogenesis assay was conducted using the In Vitro Angiogenesis Assay Kit (ECM625, Millipore Sigma) following the manufacturer's instructions. Briefly, 100 μl 10× ECMatrix Diluent buffer was mixed with 900 μl ECMatrix Gel solution on ice. The mixture was added to a 96-well plate and polymerized at 37°C for 1 h. Ten thousand HUVECs were seeded per well onto the surface of the ECMatrix gel in the EBM-2 medium. The cells were subsequently incubated at 37°C for 12 h, and tube formation was visualized using a light microscope. ImageJ was employed to skeletonize and quantify the cellular networks.

### Detection of tubulin detyrosination by liquid chromatography–tandem mass spectrometry (LC–MS/MS)

To monitor tubulin detyrosination, HEK293T cells were collected and lysed in CelLytic M cell lysis reagent (Sigma, St. Louis, MO, USA) with 1× protease inhibitor cocktail (Sigma, St. Louis, MO, USA). After separation with a 10% SDS-PAGE gel, the band in the molecular weight range of 45–55 kDa was excised and cut into small pieces, and the proteins were reduced, alkylated and digested in-gel, following previously described procedures (18). The ensuing peptide mixtures were desalted using OMIX C_18_ Tips (Agilent, Santa Clara, CA, USA) and subjected to LC-MS/MS analysis on a TSQ Altis triple-quadrupole mass spectrometer (Thermo Fisher Scientific) in the multiple-reaction monitoring mode. The transitions for the formations of b ions for the C-terminal tryptic peptide of tubulin or detyrosinated tubulin were monitored. Relative levels of the C-terminal tyrosinated or detyrosinated peptides were calculated from the peak area detected in the chromatogram for each peptide.

## RESULTS

### VEZF1 is a G-quadruplex binding protein

We previously employed a stable isotope labeling by amino acid in cell culture (SILAC)-based quantitative proteomic workflow for uncovering cellular proteins that can bind to G4 DNA (20). In that experiment, nuclear extracts of HeLa cells cultured in light and heavy (where lysine and arginine were replaced with [^13^C_6_,^15^N_2_]-l-lysine and [^13^C_6_]-l-arginine, respectively) medium were subjected to affinity pull-down using biotin-conjugated G4 DNA and the corresponding mutated single-stranded DNA incapable of folding into G4 structures, respectively, and vice versa (20). The proteomic results showed that VEZF1 could bind preferentially with G4 DNA probes derived from the promoter of *MYC* gene and human telomere (hTel) over the corresponding mutated probes (M4) that cannot fold into G4 structure (sequences of G4 and M4 probes are shown in Supplementary Table S1). Representative ESI-MS and MS/MS results for a tryptic peptide derived from VEZF1, HKLSHSDEKPFECPICNQR, are displayed in Supplementary Figure S1.

**Figure 1.**
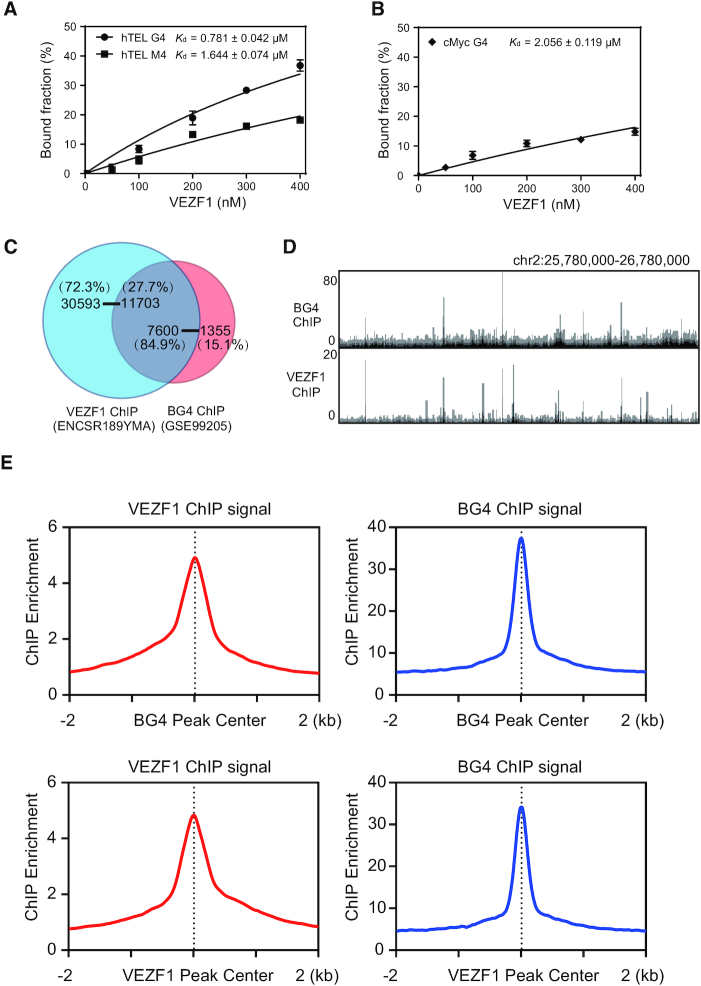
Binding of VEZF1 with G4 structures in vitro and in cells. (**A**, **B**) Quantification of *K*_d_ values for the bindings between VEZF1 and G4 DNA derived from human telomere (hTEL G4) or the promoter of *MYC* gene (cMyc G4). (**C**) A Venn diagram showing the overlap in the number of ChIP-seq peaks for VEZF1 and BG4. (**D**) A comparison of VEZF1 and BG4 ChIP-seq results in the indicated genomic region. (**E**) The average signal of VEZF1 ChIP-seq against the center of the BG4 ChIP-seq peaks, and vice versa.

Considering that the proteomic experiment may also give rise to the identification of proteins that interact indirectly with G4 DNA via protein-protein interactions, we next assessed whether VEZF1 can bind directly with G4 structures. For this purpose, we purified recombinant VEZF1 protein from HEK293T cells and measured its binding affinity towards G4 DNA probes by employing EMSA (Figure 1 and Supplementary Figure S2). Our EMSA results showed that VEZF1 binds strongly with hTEL and *MYC* G4 DNA probes, with the *K*_d_ values being 0.78 ± 0.04 and 2.06 ± 0.12 μM, respectively (Figure 1 and Supplementary Figure S2). This difference in binding affinities toward the two G4 structures suggests that the DNA sequences in the loop regions of the two G4 structures also modulate, to some extent, their interactions with VEZF1. The corresponding *K*_d_ value for binding to the mutated single-stranded DNA derived from human telomere was 1.64 ± 0.07 μM, and no binding was detectable for the mutated single-stranded DNA probe derived from the *MYC* promoter (Figure 1 and Supplementary Figure S2). These results, therefore, demonstrate that VEZF1 can bind directly and selectively with G4 structures. The higher binding affinity of VEZF1 toward the mutated single-stranded DNA derived from human telomere than the *MYC* G4 DNA probe could be attributed to other element(s) of VEZF1-DNA interaction that is independent of its ability in recognition of G4 structures.

### VEZF1 interacts with G4 structures in cells

We next asked whether VEZF1 interacts with G4 structures in cells. First, we compared the VEZF1 ChIP-seq data (NCBI GEO database: GSE136477) (21) with ChIP-seq data obtained from the use of G4 structure-specific antibody (NCBI GEO database: GSE107690) (22), to explore whether G4-enriched genomic regions are occupied by VEZF1 (Figure 1). It turned out that 7600 out of the 8955 (84.9%) confident G4 peaks were overlapped with VEZF1 ChIP-seq peaks when the size of the overlapping window is at least 1 base pair (Figure 1C, D), and 84.3% and 82.0% of confident BG4 peaks overlapped with VEZF1 ChIP-seq peaks if the sizes of the overlapping window are at least 8 and 30 bp, respectively (Supplementary Table S2). We also found that 27.2%, 27.6% and 27.7% of VEZF1 ChIP-seq peaks overlap with BG4 ChIP-seq peaks when the sizes of the overlapping window are at least 1, 8 and 30 bp, respectively (Supplementary Table S2). Signal enrichment analysis also revealed strong overlap between VEZF1 and BG4 ChIP-seq peaks (Figure 1E). Moreover, we found that the average widths for VEZF1 and BG4 ChIP-seq peaks were 596 and 226 base pairs, respectively (Supplementary Figure S3A). These results suggest the direct binding of VEZF1 with G4 structures in cells.

PDS and TMPyP4 are small-molecule ligands that can bind specifically with G4 structures (23,24). We reasoned that the binding of PDS or TMPyP4 with G4 structure may perturb the latter's interaction with VEZF1. To test this, we assessed how the chromatin localization of VEZF1 in cells is affected by PDS or TMPyP4 treatment. Chromatin fractionation followed by Western blot analysis revealed that treatment of HEK293T cells with PDS or TMPyP4 resulted in diminished localization of VEZF1 to chromatin (Supplementary Figure S3B-S3C), again substantiating the binding between VEZF1 and G4 structures in cells.

### VEZF1-G4 interaction regulates RNAP II pausing and alternative polyadenylation of VASH1

Our analysis of the genomic distributions of VEZF1 ChIP-seq peaks showed that VEZF1 occupies preferentially in introns (41.4%) and intergenic regions (28.8%) (Supplementary Figure S3D). In light of the previous finding that VEZF1 modulates RNAP II pausing (10), we also examined if VEZF1-G4 interaction elicits RNAP II pausing. Toward this end, we interrogated the previously published ChIP-seq data for VEZF1, RNAP II with serine 2 in the repetitive sequence of the C-terminal domain being phosphorylated (RNAP II-S2P), and BG4. By dividing VEZF1 ChIP-seq peaks into two groups, i.e. those with or without overlapping with BG4 ChIP-seq peaks, we found that the peaks in the first group exhibit markedly higher level of overlap with RNAP II-S2P than those in the second group (∼ 15.4% vs. 4.8%, Supplementary Table S3), underscoring that VEZF1–G4 DNA interaction promotes RNAP II pausing.

**Figure 2.**
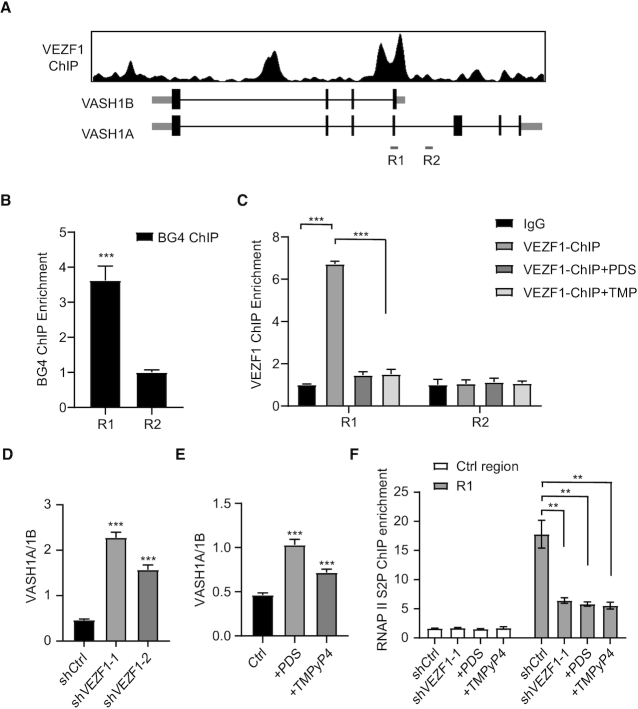
VEZF1 regulates the two different isoforms of VASH1. (**A**) VEZF1 ChIP enrichment around VASH1A region. (**B**) BG4 ChIP-qPCR confirmed the formation of G4 structure at the junction of VASH1A and VASH1B in HEK293T cells. (**C**) VEZF1 ChIP-qPCR showing the diminished occupancies of VEZF1 at the junction of VASH1A and VASH1B in HEK293T cells treated with PDS or TMPyP4 (TMP). (D, E) The changes in VASH1A/VASH1B ratio in HEK293T cells upon genetic knockdown of VEZF1 (**D**), or after PDS or TMPyP4 treatment (**E**). (**F**) RNAP II S2P ChIP-qPCR results showing the occupancy of RNAP II S2P at the VASH1A-VASH1B junction in HEK293T cells after shRNA-mediated knockdown of VEZF1, or after treatment with PDS or TMPyP4.

We next investigated whether VEZF1 is involved in modulating the relative levels of the two isoforms of VASH1, i.e. VASH1A and VASH1B (16,25), where the latter contains only the N-terminal 176 amino acid residues of the former (Figure 2A) (16). Our analysis of the VEZF1 ChIP-seq data showed that VEZF1 binds to the region of the DNA situated at the junction of the two VASH1 isoforms (Figure 2A and Supplementary Figure S4). We also found that the junction region contains a number of guanine repeats, which is highly susceptible to G4 structure formation (Supplementary Figure S4) (26).

We next examined the formation of G4 structures from two sequences derived from junction region in vitro (Supplementary Figure S5). Circular dichroism (CD) spectra confirmed the formation of G4 structures from these sequences, but not from the corresponding sequences with the central guanine residues in the G-tracks being mutated to thymines (Supplementary Figure S5). Moreover, our BG4 ChIP-qPCR experiment revealed the presence of G4 structures in this region in HEK293T cells (Figure 2B).

Our EMSA results showed that VEZF1 protein can bind directly with the two G4 structures derived from the junction region, with the *K*_d_ values being 0.649 and 0.730 μM, respectively (Supplementary Figure S6). Mutations of central guanines in the G tracks to thymines again abrogate the binding with VEZF1 (Supplementary Figure S6). Furthermore, treatment of cells with a G4-stabilizing small molecule, PDS or TMPyP4, attenuates the binding of VEZF1 towards this region, but not a nearby control region (Figure 2C), substantiating that VEZF1’s binding to the region depends on the presence of G4 structure.

We next investigated the impact of VEZF1 on alternative polyadenylation of VASH1 mRNA by measuring the relative levels of VASH1A and VASH1B mRNA after shRNA-mediated knockdown of VEZF1 (Figure 2D and Supplementary Figure S4). Our results showed that genetic depletion of VEZF1 in HEK293T cells with two different sequences of shRNA could markedly increase the VASH1A/VASH1B ratio (Figure 2D), and similar observations were made for cells treated with PDS or TMPyP4 (Figure 2E). These results demonstrated the involvement of VEZF1–G4 interaction in regulating the two isoforms of VASH1 mRNA.

On the basis of the above findings, we hypothesized that the interaction between VEZF1 and G4 structure at the junction of VASH1A and VASH1B elicits RNAP II pausing at the site, thereby triggering alternative polyadenylation and generation of the short isoform of VASH1 (i.e. VASH1B). To test this hypothesis, we performed ChIP-qPCR experiments to examine the occupancy of RNAP II-S2P at the junction region of VASH1A and VASH1B (Figure 2F). Our results revealed that RNAP II-S2P enrichment is attenuated upon knockdown of VEZF1, or upon treatment with PDS or TMPyP4 (Figure 2F).

To further confirm the involvement of VEZF1-G4 binding in RNAP II transcription pausing and alternative polyadenylation, we conducted an in vitro transcription assay using a template harboring a G4-forming sequence derived from the junction region of VASH1A and VASH1B, and the corresponding mutated sequence that cannot fold into G4 structure (Supplementary Figure S7). Our results showed that the G4-forming sequence derived from VASH1, but not the mutated sequence, led to transcriptional arrest at the junction region and resulted in the formation of short transcript(s) (Supplementary Figure S7). Hence, our results indicate that VEZF1 binding with G4 structure at the junction of VASH1A and VASH1B contributes to RNAP II pausing at the locus, thereby giving rise to alternative polyadenylation and promoting the generation of the VASH1B over VASH1A isoform.

### VEZF1-mediated alternative polyadenylation of VASH1 mRNA influences tubulin detyrosination

**Figure 3.**
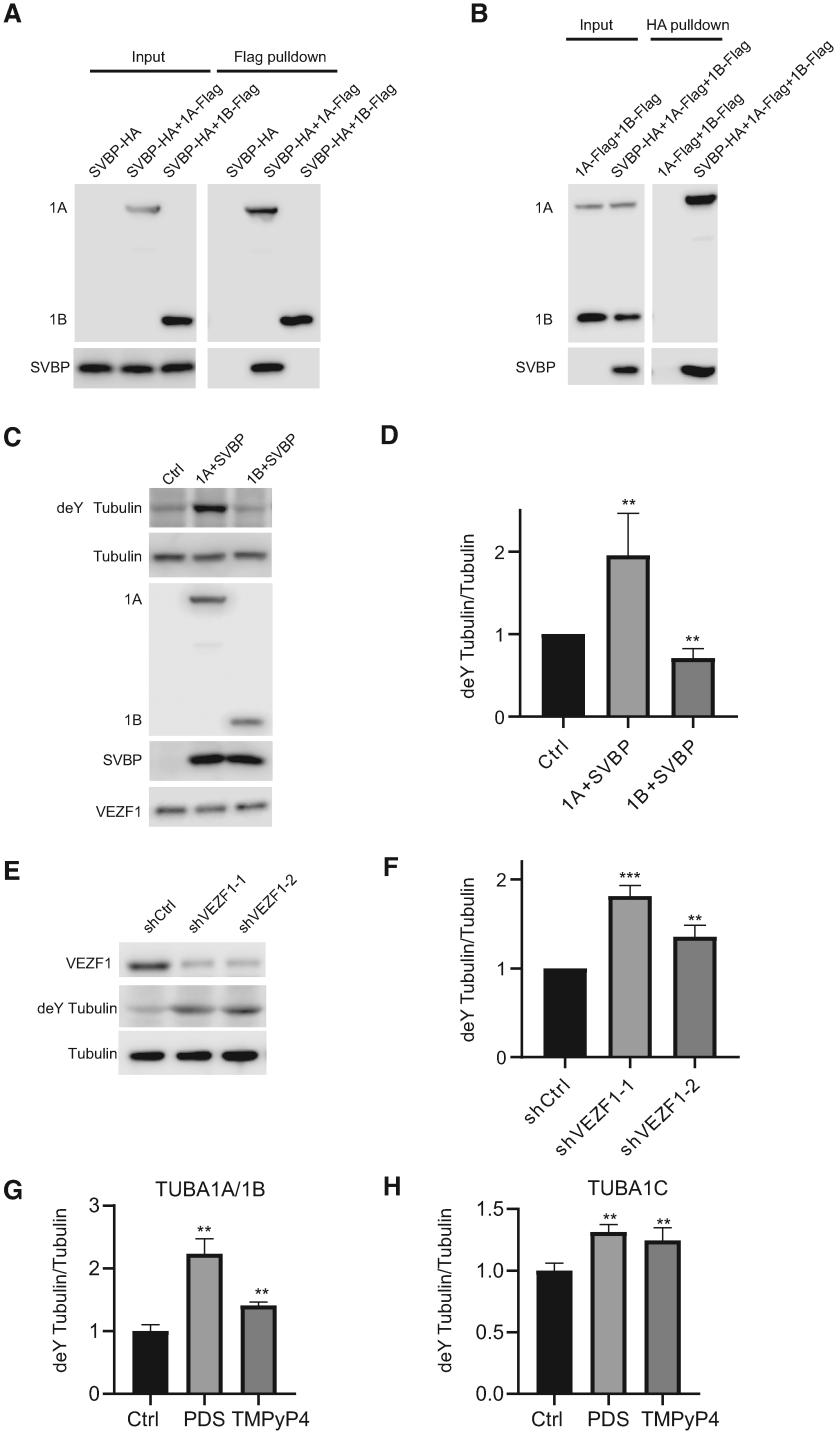
VASH1A and VASH1B interact with SVBP to regulate tubulin detyrosination. (**A, B**) Reciprocal immunoprecipitation followed by immunoblot analysis confirmed the interaction between SVBP and VASH1A, but not between SVBP and VASH1B. (**C, D**) VASH1A-SVBP facilitates tubulin detyrosination, whereas VASH1B-SVBP inhibits tubulin detyrosination. (**E–H**) Detyrosination of tubulin A1B and A1C is elevated in HEK293T cells upon shRNA-mediated knockdown of VEZF1 (E, F), or after treatment with PDS or TMPyP4 (G, H). Tubulin detyrosination was quantified with LC-MRM analysis (Supplementary Figure S9).

VASH1 and VASH2, when form a heterodimer with SVBP, were recently discovered as the long-sought carboxypeptidase for tubulin detyrosination (12,13). Hence, we also asked whether dynamic detyrosination of tubulins could be affected by VEZF1–G4 DNA interaction. First, we examined the heterodimer formation between SVBP and the two isoforms of VASH1 (Figure 3A, B). Reciprocal pull-down experiments revealed that VASH1A, but not VASH1B, could interact with SVBP (Figure 3A, B). Simultaneous overexpression of VASH1A with SVBP in cells results in elevated detyrosination of tubulin, whereas concurrent overexpression of VASH1B with SVBP leads to a decreased level of detyrosinated tubulin (Figure 3C, D, Supplementary Figure S8). This result demonstrates the distinct roles of VASH1A and VASH1B in tubulin detyrosination.

Knockdown of VEZF1 again results in elevated VASH1A/VASH1B ratio and augments the level of detyrosinated tubulin in cells (Figure 3E-F). Similar findings were made for cells treated with PDS or TMPyP4 (Figure 3G, H). Together, our results revealed that VEZF1–G4 interaction modulates the alternative polyadenylation of VASH1 and regulates tubulin detyrosination in cells.

### VEZF1-mediated alternative polyadenylation modulates angiogenesis

**Figure 4.**
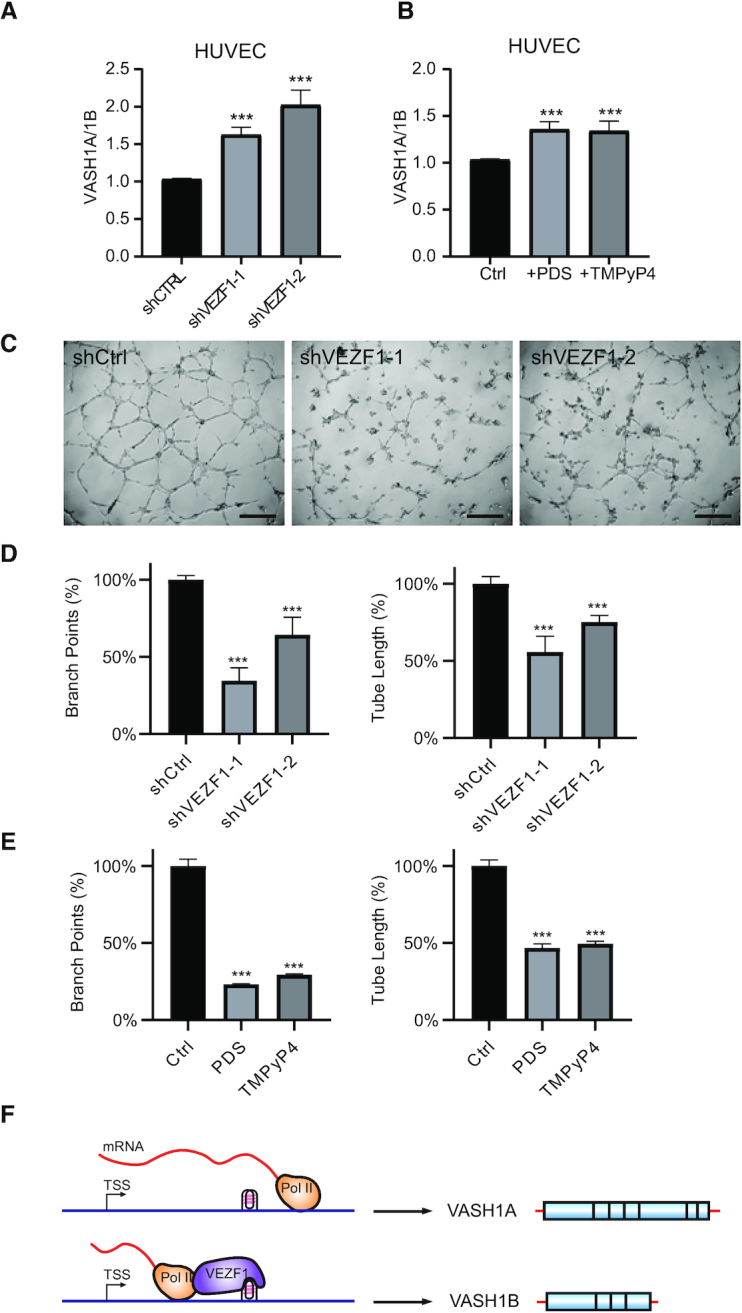
VEZF1 regulates angiogenesis in HUVECs. (A, B) The alterations in VASH1A/VASH1B transcript ratio in HUVECs upon shRNA-mediated knockdown of VEZF1 (**A**), or after treatment with PDS or TMPyP4 (**B**). (**C**) Angiogenesis in HUVECs after knockdown of VEZF1. Scale bar: 0.2 mm. (D, E) Quantification of branch points and tube length after genetic knockdown of VEZF1 (**D**), or after treatment with PDS or TMPyP4 (**E**), as obtained from angiogenesis assay. (**F**) A schematic diagram showing the regulation of the two VASH1 isoforms by VEZF1-mediated binding of G4 structure.

Previous reports showed that the two isoforms of VASH1 play distinct roles in angiogenesis (16,25). Hence, we asked whether the VEZF1-mediated alternative polyadenylation of VASH1 is involved in angiogenesis. We found that, similar to what were observed for HEK293T cells (Figure 4), genetic depletion of VEZF1, or treatment with PDS or TMPyP4, confers elevated ratios of VASH1A/VASH1B in HUVECs (Figure 4A, B). In addition, our angiogenesis assay with the use of HUVECs showed that knockdown of VEZF1, or treatment of these cells with PDS or TMPyP4, elicits diminished branch point number and the total tube length (Figure 4C–E and Supplementary Figure S9). Together, these results substantiated that VEZF1–G4 DNA interaction and the ensuing alternative polyadenylation of VASH1 modulate angiogenesis.

## DISCUSSION

VEZF1 is known to interact with G-rich DNA sequences. For instance, the chicken ortholog of human VEZF1 (i.e. BGP1) was shown to bind with poly(dG) sequences (27,28). In addition, a previous ChIP-seq study revealed >30 000 VEZF1 binding sites in chromatin; although no consensus binding sequences emerged from the ChIP-seq data, the survey revealed the enrichment of simple repeats, including different variants of G track-containing repeats, in the VEZF1-binding targets (10). Here, our unbiased quantitative proteomic screening and *in-vitro* biochemical experiments unveiled VEZF1 as a protein that can bind directly with G4 DNA. Moreover, analysis of the VEZF1 ChIP-seq data and BG4 ChIP-seq data together showed that the majority of the G4 loci in chromatin overlap with VEZF1-binding sites (Figure 2 and Supplementary Table S2). Thus, our results unveiled a novel and crucial molecular determinant for VEZF1-DNA recognition, i.e. through binding of G4 structures.

Our results also demonstrated that VEZF1 regulates alternative polyadenylation of VASH1, and this regulation involves VEZF1–G4 DNA interaction. Mouse VEZF1 is highly expressed in vascular endothelium during early development, and it is also expressed in neuronal and mesodermal tissues in embryos (29). In addition, several transcriptional targets of VEZF1 are known to be involved in angiogenesis (6–9). Moreover, a previous study showed a strong correlation between VEZF1 binding and enrichment of RNAP II-S2P, which reflects RNAP II pausing (10). Our analysis of previously published ChIP-seq data revealed much more pronounced RNAP II pausing at those VEZF1-binding loci that assume G4 structures than those that do not (Supplementary Table S3), suggesting that VEZF1-G4 interaction contributes to RNAP II pausing.

We also found that VEZF1 can bind directly with G4-forming sequences located in the junction region between VASH1A and VASH1B (Supplementary Figure S6), and this binding modulates the alternative polyadenylation of VASH1 mRNA (Supplementary Figure S7). The role of VEZF1–G4 binding in this process is manifested by the observation that both treatment with G4-stabilizing small molecules (i.e. PDS and TMPyP4) and genetic depletion of VEZF1 led to increased ratio of VASH1A/VASH1B mRNA (Figure 2). Along this line, pausing of RNAP II elongation at G-rich sequences was found to activate polyadenylation at upstream sites to yield short polyadenylated transcripts (30). Results from our in-vitro transcription assay also showed that G4 sequences from the junction region of VASH1A and VASH1B can result in transcription stalling, which was abolished when the G4 sequence is mutated to one that can no longer assemble into G4 structures (Supplementary Figure S7). Hence, we reason that RNAP II pausing at the G4 site located at the junction of VASH1A and VASH1B could be alleviated by genetic depletion of VEZF1 or upon displacement of VEZF1 from DNA by competitive binding with G4-stabilizing small molecules, thereby giving rise to augmented transcriptional read-through and increased VASH1A/VASH1B ratio (Figure 4F). This modulation of RNAP II transcription pausing by G4 DNA is in line with the notion that mRNA splicing and polyadenylation can occur co-transcriptionally (31). In this context, it is of note that a G4 structure located in an intron region was previously shown to regulate alternative splicing of mRNA of *TP53* gene (32). It will be important to examine, in the future, how disruption of VEZF1-G4 binding modulates alternative polyadenylation at the genome-wide scale.

We also illustrated a role of VEZF1 in the alternative polyadenylation of VASH1 in HUVECs, which modulates the tubulin detyrosinase activity of VASH1 and angiogenesis in HUVECs. In particular, we found that genetic depletion of VEZF1 or treatment with G4-stabilizing small molecules leads to increased detyrosination activities (Figure 3). This can be rationalized from the differences in amino acid sequences in the C-terminal region of the two splicing variants of VASH1. In this vein, VASH1A and VASH1B share the first N-terminal 176 amino acids (16), and recent X-ray crystal structures of VASH1-SVBP complexes revealed that Cys169, His204 and Ser221 form a non-classical catalytic triad (33–35). Hence, the absence of essential residues for catalysis (i.e. His204 or Ser221) renders VASH1B inactive in tubulin detyrosination. We also found that only VASH1A, but not VASH1B, forms a complex with SVBP (Figure 3).

The disruption of angiogenesis in HUVECs upon genetic depletion of VEZF1 or its displacement from chromatin by G4-stabilizing small molecules, which confers an elevated ratio of detyrosinase-competent VASH1A over the incompetent VASH1B, is consistent with the notion that homeostasis of dynamic tyrosination/detyrosination of α- and β-tubulins is crucial for angiogenesis (12,13). Viewing that the VASH1-SVBP-mediated tubulin detyrosination is also indispensable in neuron differentiation (12), it can be envisaged that the VEZF1-modulated alternative polyadenylation also assumes important role in this process.

To summarize, we discover VEZF1 as a G quadruplex-binding protein, illustrate the role of this binding in regulating the alternative polyadenylation of VASH1 mRNA, and reveal the distinct functions of the two isoforms of VASH1 mRNA in angiogenesis. Therefore, our results support that the VEZF1- and VASH1-mediated regulatory pathways of angiogenesis are functionally connected. Our work also unveils a novel function of G-quadruplex DNA (i.e. modulation of RNAP II pausing) and uncovers VEZF1 as a key molecular player in this process. Moreover, our study shows that targeting G quadruplex DNA with small-molecule drugs (36) may also disrupt some essential functions in cells and elicit unintended consequences; thus, additional research toward understanding more completely the interactions between G quadruplex DNA and proteins will benefit future drug design in this area.

## DATA AVAILABILITY

The ChIP-seq data for VEZF1 were obtained from NCBI GEO database with accession numbers of GSE136477. The G4 ChIP-seq data were obtained from NCBI GEO database with accession numbers of GSE107690. The RNAP II-S2P ChIP-seq data were obtained from NCBI GEO database with accession numbers of GSM935402.

## Supplementary Material

gkaa1092_Supplemental_File
