# Supplementary material for: VEZF1–guanine quadruplex DNA interaction regulates alternative polyadenylation and detyrosinase activity of VASH1
Source: Nucleic Acids Res. 2020 Nov 24;48(21):11994–2003. doi: 10.1093/nar/gkaa1092 (PMC7708047; doi:10.1093/nar/gkaa1092)

Supplementary Materials for

"VEZF1-Guanine Quadruplex DNA Interaction Regulates Alternative Polyadenylation and Detyrosinase Activity of VASH1"

by

Lin Li, Preston Williams, Zi Gao and Yinsheng Wang*

Department of Chemistry, University of California, Riverside, CA 92521, USA

*Corresponding Author: Yinsheng Wang, Tel.: (951) 827-2700; Fax: (951) 827-4713;

E-mail: Yinsheng.Wang@ucr.edu

Table of Contents:

Contents	Page #
**Table S1.** Primers and other DNA sequences used in this study.	S-3
**Table S2.** Analysis of peak overlapping between VEZF1 ChIP-seq and BG4 ChIP-seq data with different size windows.	S-4
**Table S3.** Analysis of overlapping between VEZF1 ChIP-seq peaks and ChIP-seq peaks for serine 2 phosphorylated form of RNA Polymerase II (RNAP II-S2P).	S-5
**Figure S1.** Positive-ion electrospray ionization-mass spectra (ESI-MS) showing the preferential binding of VEZF1 to G4 structure formed from the sequence derived from human telomere region.	S-6
**Figure S2.** EMSA results showing the direct binding of VEZF1 with G4 DNA.	S-7
**Figure S3.** Statistical analysis of VEZF1 ChIP-seq and BG4 ChIP-seq data.	S-8
**Figure S4.** The DNA sequence of the VEZF1 binding peak at the junction of the VASH1A and VASH1B and RT-qPCR for assessing the knockdown efficiency of VEZF1 with shRNA.	S-9
**Figure S5.** The G-rich sequences derived from the VASH1A-VASH1B junction can form G4 structure in vitro.	S-10

**Figure S6.** In vitro binding of VEZF1 with G4 structures derived from the junction of VASH1A and VASH1B.	S-11
**Figure S7.** Transcription pausing caused by G4 structure from the sequence derived from the junction of VASH1A and VASH1B.	S-12
**Figure S8.** Quantification of tubulin and detyrosinated tubulin with LC-MS/MS analysis in the MRM mode.	S-13
**Figure S9.** Representative images showing the results obtained from angiogenesis assay in HUVECs after treatment with PDS or TMPyP4.	S-14

Supplemental Table S1. Primers or sequences used in this study.

**G4 and mutated probes**	
Name	Sequence
MYC G4	5′-TAMRA-TGA GGG TGG GGA GGG TGG GGA-3′
MYC M4	5′-TAMRA-TGA GGG TGA GGA GTG TGG GGA-3′
hTEL G4	5′-TAMRA-AAA GGG TTA GGG TTA GGG TTA GGG AA-3′
hTEL M4	5′-TAMRA-AAA GGG TTA GTG TTA GTG TTA GGG AA-3′
**Sequences used for shRNA**	
shVEZF1-1 targeted sequence	5′-GTACTTTGGAACAGTACAAAT-3′
shVEZF1-2 targeted sequence	5′-CATTGCTTCCAATACCAATAA-3′
**Primers used for real-time quantitative PCR**	
VEZF1-F	5′-TGCAGTGTTTGTGGGAAAGG-3′
VEZF1-R	5′-GCACGTTTGGCATTTGAAGG-3′
VASH1A-F	5′-AGTTCAGGATCAGGCTGCTT-3′
VASH1A –R	5′-CTGTCCCTGGCAGACTTACA-3′
VASH1B –F	5′-TGCTACCTTCCCAGGCTTAC-3′
VASH1B –R	5′-GACACTGTGCCCTGATTTGG-3′
GAPDH-F	5′-TTCGACAGTCAGCCGCATCTTCTT-3′
GAPDH-R	5′-CAGGCGCCCAATACGACCAAATC-3′
ChIP-PCR-R1-F	5′-CCAGAGCTCTTTGGAGCAAG-3′
ChIP-PCR-R1-R	5′-GATAGGAAGGAGGCCCACTC-3′
ChIP-PCR-R2-F	5′-ACAACATCATGAACTCCTGTGC-3′
ChIP-PCR-R2-R	5′-GGCAGGGTGGAAGAGATGAA-3′

Supplemental Table S2. Analysis of peak overlapping between VEZF1 ChIP-seq and BG4 ChIP-seq data with different size windows.

A

Overlap size windows	Total number of BG4 ChIP-seq peaks	Number (percentage) of BG4 Peaks Overlapped with VEZF1 ChIP-seq peaks	No overlap
≥ 1 bp	8955	7600 (84.9%)	1355
≥ 8 bp	8955	7552 (84.3%)	1403
≥ 30 bp	8955	7344 (82.0%)	1611

B

Overlap size windows	Total number of VEZF1 ChIP-seq peaks	Number (percentage) of VEZF1 Peaks Overlapped with BG4 ChIP-seq peaks	No overlap
≥ 1 bp	42296	11703 (27.7%)	30593
≥ 8 bp	42296	11664 (27.6%)	30632
≥ 30 bp	42296	11524 (27.2%)	30772

Supplemental Table S3. Analysis of overlapping between VEZF1 ChIP-seq peaks and ChIP-seq peaks for serine 2 phosphorylated form of RNA Polymerase II (RNAP II-S2P), where the VEZF1 ChIP-seq peaks were divided into two groups, one with overlap with BG4 ChIP-seq peaks, and the other without. Overlap size windows refer to the number of base pairs overlapped between VEZF1 and BG4 ChIP-seq peaks.

Overlap size windows	VEZF1 ChIP-seq Total peaks	VEZF1 Peaks Overlapped with BG4 ChIP-seq peaks		VEZF1 Peaks with no Overlap with BG4 ChIP-seq Peaks	
		Number (%) of Peaks Overlapped with RNAP II-S2P	No overlap	Number (%) of Peaks Overlapped with RNAP II-S2P	No overlap
≥ 1 bp	42296	1565 (15.4%)	10138	1400 (4.8%)	29193
≥ 8 bp	42296	1561 (15.4%)	10103	1391 (4.8%)	29241
≥ 30 bp	42296	1536 (15.4%)	9988	1384 (4.7%)	29388

Figure S1. Positive-ion electrospray ionization-mass spectra (ESI-MS) showing the preferential binding of VEZF1 to G4 structure formed from the sequence derived from human telomere region. Shown are the ESI-MS for the light (monoisotopic peak at *m/z* 596.28) and heavy (monoisotopic peak at *m/z* 601.79) lysine and arginine-containing tryptic peptide HKLSHSDEKPFECPICNQR from VEZF1 (A-B). In (A), the biotin-labeled G4 DNA probe and mutated single-stranded DNA probe were incubated with the nuclear extracts of light- and heavy-labeled HeLa cells, respectively. The opposite incubation was conducted for generating the results in (B). MS/MS of the $[M+4H]^{4+}$ ions of the light (*m/z* 596.28) and heavy (*m/z* 601.79) lysine and arginine-containing tryptic peptide HKLSHSDEKPFECPICNQR from VEZF1 are shown (C-D).

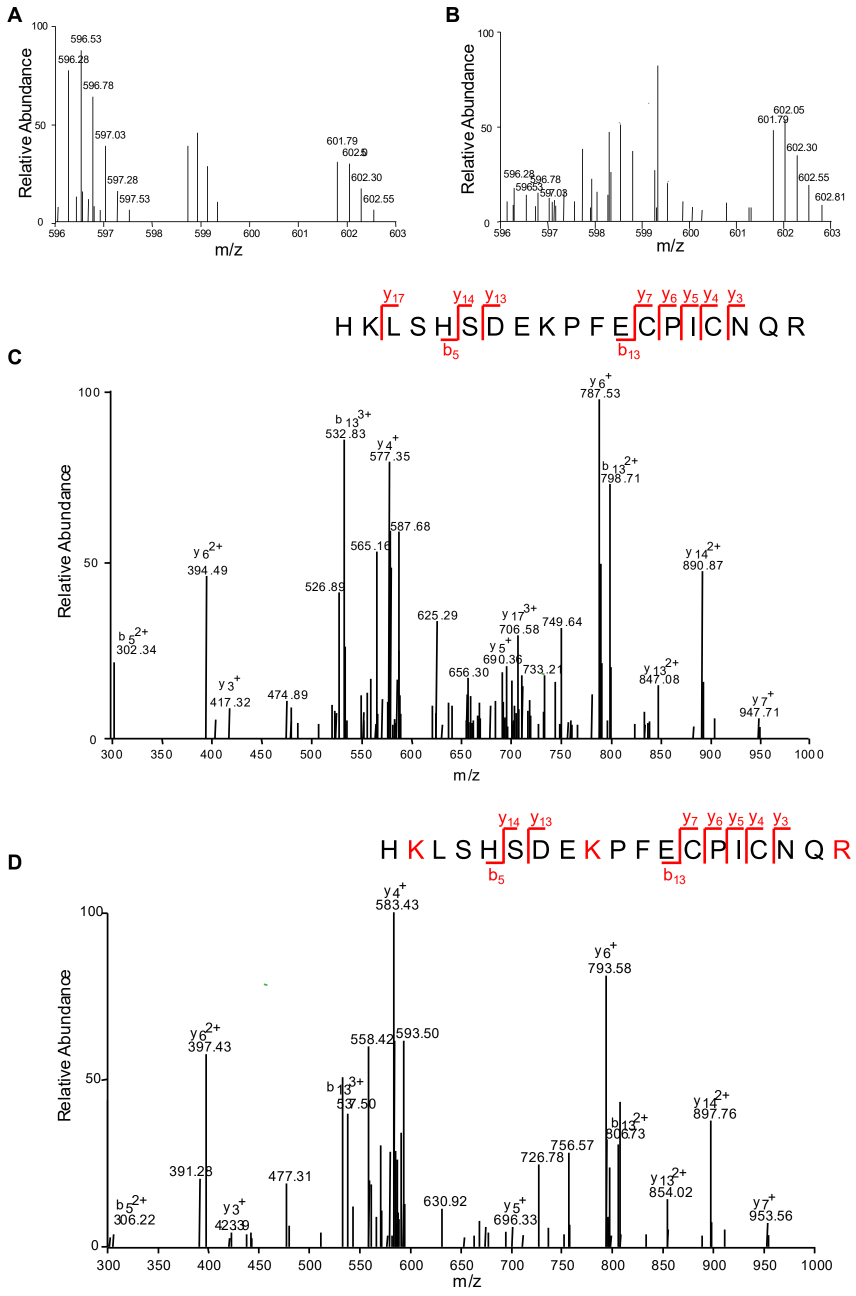

Figure S2. EMSA results showing the direct binding of VEZF1 with G4 DNA. Shown are the EMSA gel images for monitoring the bindings of VEZF1 with the G4 structures derived from human telomere (hTEL G4) (A) and the promoter of *MYC* gene (*MYC* G4) (B), and the corresponding mutated sequences that cannot fold into G4 structures (i.e. hTEL M4 and *MYC* M4).

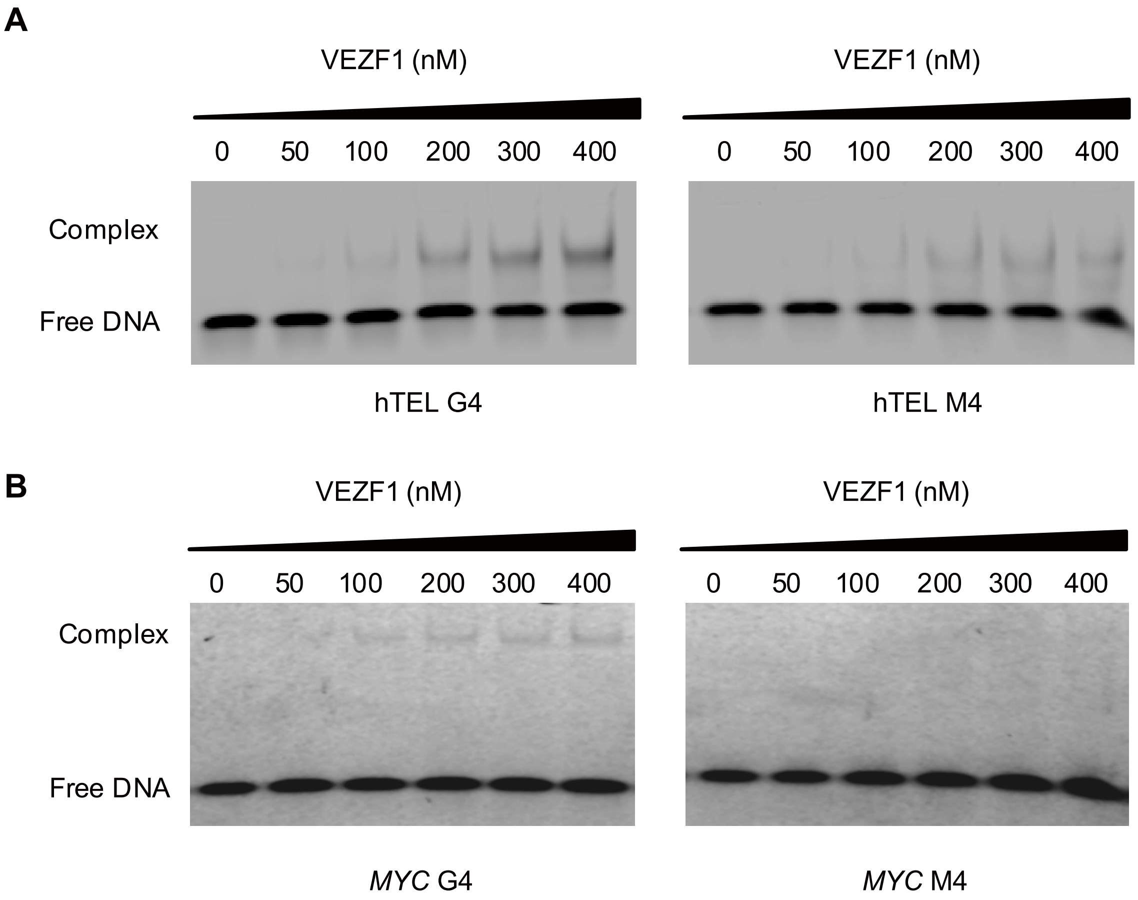

Figure S3. Statistical analysis of VEZF1 ChIP-seq and BG4 ChIP-seq data. (A) Peak width distributions of VEZF1 and BG4 based on their ChIP-seq data. (B-C) Chromatin localization of VEZF1 in HEK293T cells at different time intervals following PDS (B) or TMPyP4 (C) treatment. (D) Genomic distributions of VEZF1 ChIP-seq peaks.

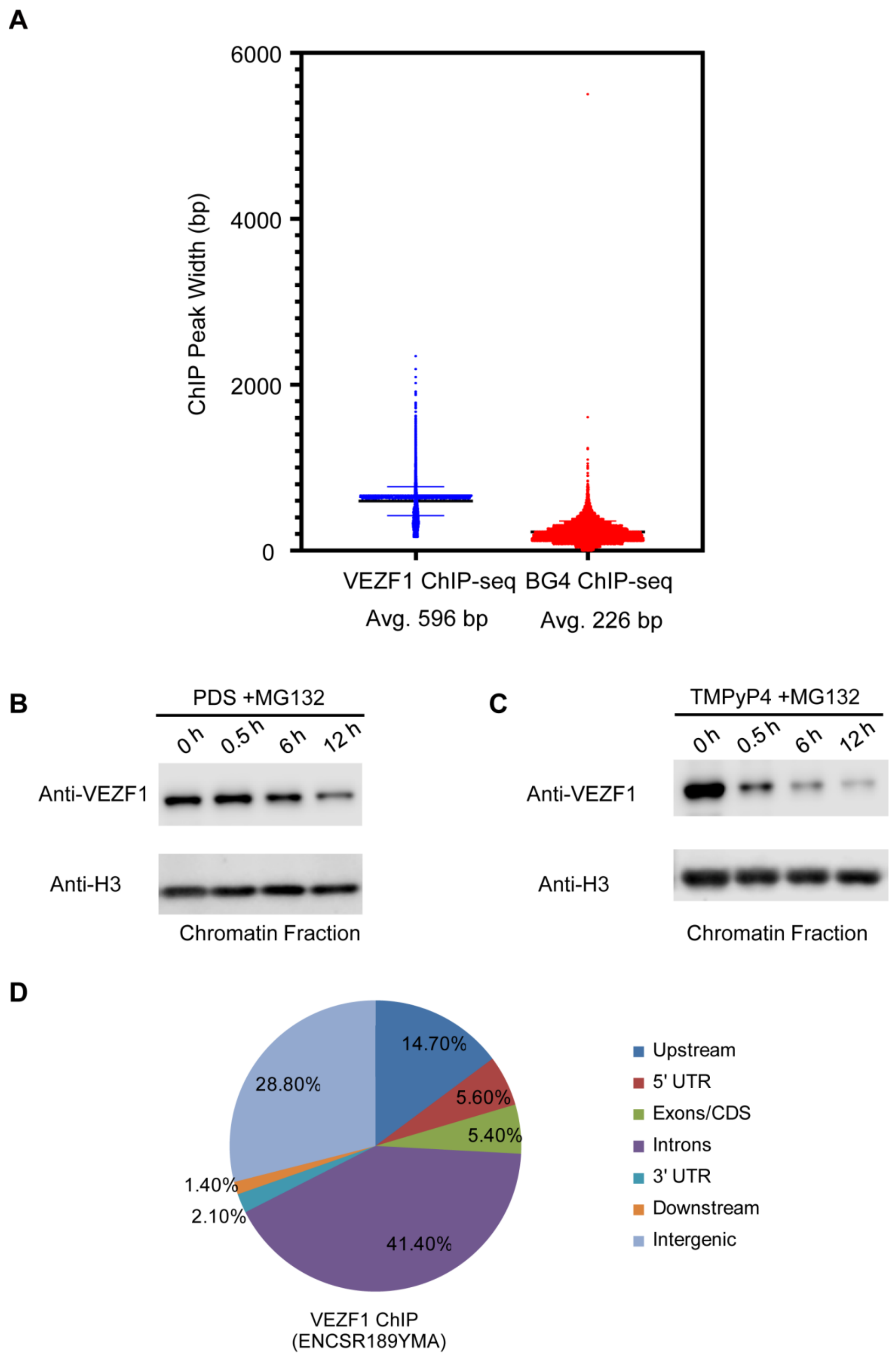

Figure S4. (A) The DNA sequence of the VEZF1 binding peak at the junction of VASH1A and VASH1B. The guanine repeats are labeled in red. (B) The knockdown efficiencies of VEZF1 shRNAs were confirmed by RT-qPCR.

A

CCTCCCCC GACTGCCTCGCAGAAGGCCCC AGCCTGGTGGCTGGAGAGAAG
TTGCCCCC AGCTTCTGCTGCCCTCCCCCAGAGCTCTTT GGAGCAAGAGCCA
GGCGTGGGAGGTGGAAGTGAAAGAAGGGAAGAGCAGGGAGAGTGGGCCT
CCTTCCTATCAGCAGTGGCCGCCTCTTTCCCACCCCCTCAGGCTCCAGACT
TCCGTCCGTCGGGAGTGTGGAGGGAGGTGCTGGGCCTGAGCTGAGAGCA
GCTTAGGGGGCAGCCTCTGTCCTTCTAGCATTTCTAGTCCACCCTGCCCCCT
CCTTCTCACATTCCCCTGGAGGGGCTGGAGGCGCTGTCCTTACTGACCTGC
CTCCCTTTCCCACAGGCTGATGGAC

B

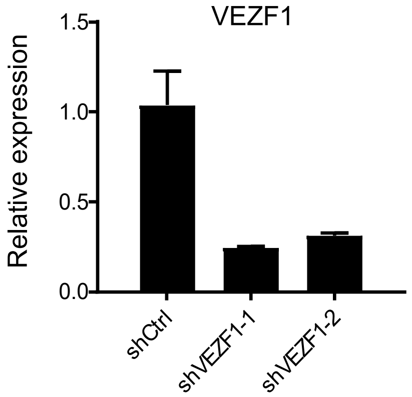

Figure S5. The G-rich sequences derived from the VASH1A-VASH1B junction can form G4 structure in vitro. (A) The G-rich sequences derived from the junction of VASH1A and VASH1B and their mutated counterparts. (B) Circular dichroism (CD) spectra showing the formation of G4 structures for the sequences derived from the wild-type sequence, but not those from the corresponding mutated sequences.

A

R1G4-1 GTGGGAGGTGGAAGTGAAAGAAGGGAAGAGCAGGGAGAGTGGGCC

R1M4-1 GTGTGAGGTGGAAGTGAAAGAAGTGAAGAGCAGTGAGAGTGTGCC

R1G4-2 TCGGGAGTGTGGAGGGAGGTGCTGGGCCTGAGCTGAGAGCAGCTTAGGGGGCA

R1M4-2 TCGTGAGTGTGGAGTGAGGTGCTGTGCCTGAGCTGAGAGCAGCTTAGTTTGCA

B

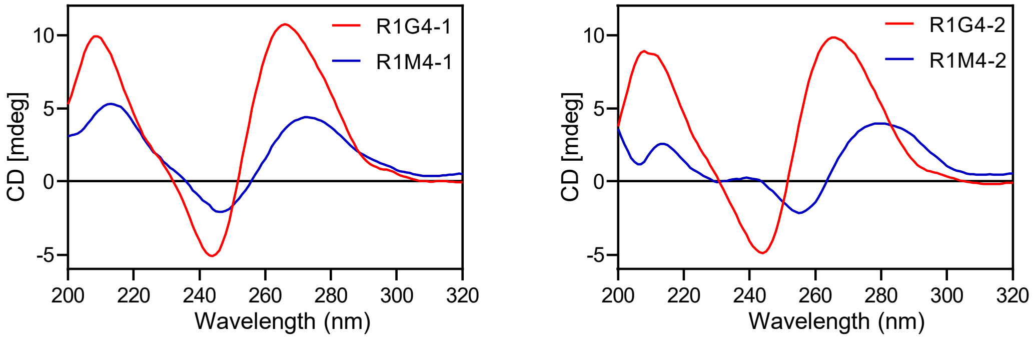

Figure S6. In vitro binding of VEZF1 with G4 structures derived from the junction of VASH1A and VASH1B. (A-B) EMSA showing the binding of VEZF1 with G4 sequences derived from the junction of VASH1A and VASH1B, but not with the mutated sequences that cannot fold into G4 structures. (C-D) Quantification of the dissociation constants (K_d) for the binding between VEZF1 and the two G4 sequences derived from the junction of VASH1A and VASH1B.

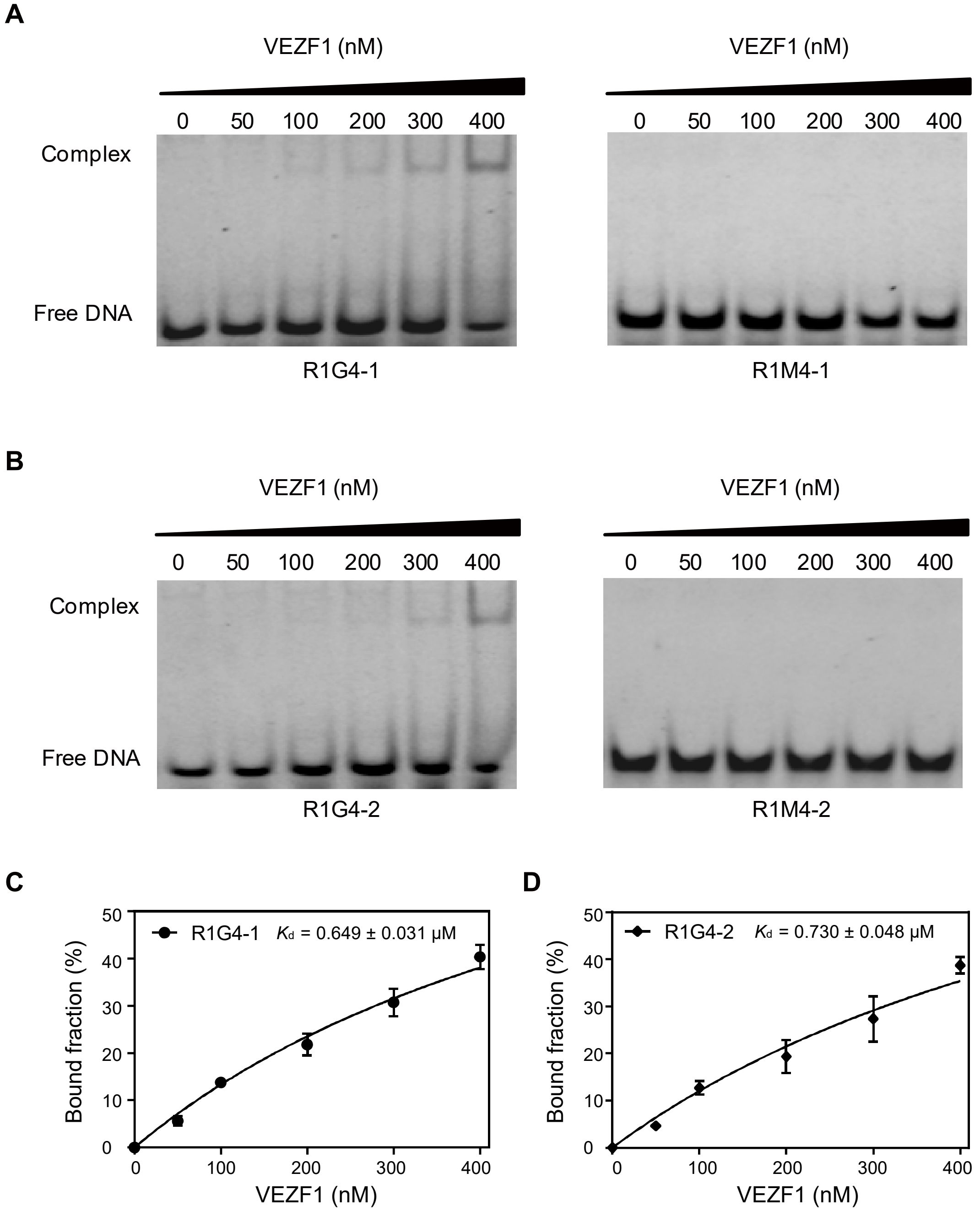

Figure S7. Transcription pausing caused by G4 structure from the sequence derived from the junction of VASH1A and VASH1B. (A) G4 sequence from VASH1 and its mutated counterpart inserted into the template for in-vitro transcription assay. (B) Schematic diagrams showing the two DNA templates employed for the in vitro transcription assay. (C) In vitro transcription assay showing the transcriptional pausing induced by G4 sequences derived from the junction of VASH1A and VASH1B, but not by the corresponding mutated sequence that cannot fold into G4 structure.

A

G4 sequences from VASH1

CCTCCCCCGACTGCCTCGCAGAAGGCCCCAGCCTGGTGGCTGGAGAGAAGTTGC
CCCCAGCTTCTGCTGCCCTCCCCCAGAGCTCTTTGGAGCAAGAGCCAGGCGTGG
GAGGTGGAAGTGAAAGAAGGGAAGAGCAGGGAGAGTGGGCCTCCTTCCTATCAG
CAGTGGCCGCCTCTTTCCCACCCCCTCAGGCTCCAGACTTCCGTCCGTCGGGAG
TGTGGAGGGAGGTGCTGGGCCTGAGCTGAGAGCAGCTTAGGGGGCAGCCTCTG
TCCTTCTAGCATTTCTAGTCCACCCTGCCCCCTCCTTCTCACATTCCCCTGGAGGG
GCTGGAGGCGCTGTCCTTACTGACCTGCCTCCCTTTCCCACAGGCTGATGGAC

Mutated G4 sequences from VASH1

CCTCTTTCGACTGCCTCGCAGAAGGCTCCAGCCTGGTGGCTGGAGAGAAGTTGC
TCCCAGCTTCTGCTGCTCTCTCCCAGAGCTCTTTGGAGCAAGAGCCAGGCGTGT
GAGGTGGAAGTGAAAGAAGTGAAGAGCAGTGAGAGTGTGCCTCCTTCCTATCAG
CAGTGGCCGCCTCTTTCTCACTCCCTCAGGCTCCAGACTTCCGTCCGTCGTGAGT
GTGGAGTGAGGTGCTGTGCCTGAGCTGAGAGCAGCTTAGTTTGCAGCCTCTGTC
CTTCTAGCATTTCTAGTCCACTCTGCTTTCTCCTTCTCACATTCTCCTGGAGTGGCT
GGAGGCGCTGTCCTTACTGACCTGCCTCTCTTTCTCACAGGCTGATGGAC

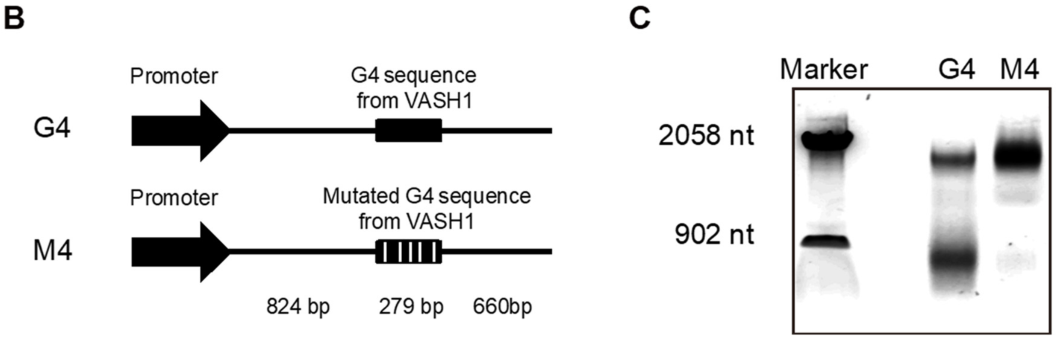

Figure S8. Quantification of tubulin and detyrosinated tubulin with LC-MS/MS analysis in the MRM mode. (A) Shown are selected-ion chromatograms for the C-terminal peptides from tyrosinated or detyrosinated TUBA1C and TUBA1A/1B. The peaks represent the corresponding peptides were labeled with red dot. (B-C) The MS/MS showing the monitored b ions from the C-terminal peptides from tyrosinated or detyrosinated TUBA1C (B) and TUBA1A/1B (C).

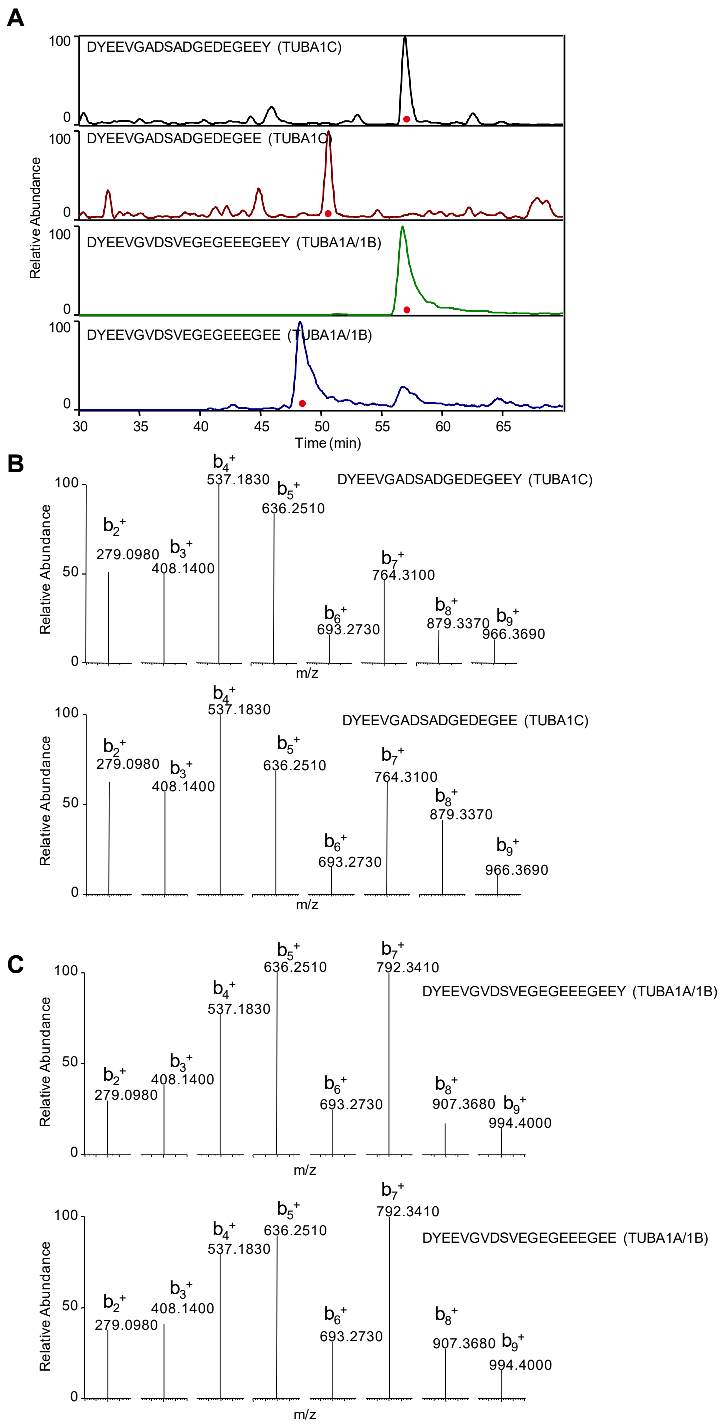

Figure S9. Representative images showing the results obtained from angiogenesis assay in HUVECs after treatment with PDS or TMPyP4. Scale bar = 0.2 mm.

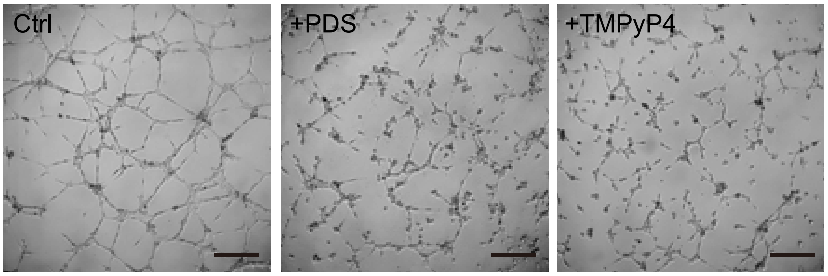

Supplement: gkaa1092_Supplemental_File [file gkaa1092_supplemental_file.pdf]
